# Rotavirus Reverse Genetics Systems and Oral Vaccine Delivery Vectors for Mucosal Vaccination

**DOI:** 10.3390/microorganisms13071579

**Published:** 2025-07-04

**Authors:** Jun Wang, Songkang Qin, Kuanhao Li, Xin Yin, Dongbo Sun, Jitao Chang

**Affiliations:** 1 State Key Laboratory for Animal Disease Control and Prevention, Harbin Veterinary Research Institute, Chinese Academy of Agricultural Sciences, Harbin 150069, China; wj057596@163.com (J.W.); songkang.qin@doct.uliege.be (S.Q.); 18263737331@163.com (K.L.); yinxin@caas.cn (X.Y.); 2 College of Animal Science and Veterinary Medicine, Heilongjiang Bayi Agricultural University, No. 5 Xinfeng Road, Sartu District, Daqing 163319, China; 3 Laboratory of Molecular and Cellular Epigenetics, Grappe Interdisciplinaire de Génoprotéomique Appliquée, University of Liège, 4000 Liège, Belgium; 4 Molecular Biology, Teaching and Research Center, University of Liège, 5030 Gembloux, Belgium; 5 Institute of Western Agriculture, Chinese Academy of Agricultural Sciences, Changji 831100, China

**Keywords:** rotavirus, reverse genetics, recombinant rotavirus, transduction vector, mucosal immunity

## Abstract

Mucosal immunization represents a promising strategy for preventing enteric infections. Rotavirus (RV), a leading gastrointestinal pathogen distinguished by its remarkable stability and segmented double-stranded RNA genome, has been engineered into a versatile oral vaccine vector through advanced reverse genetics systems. The clinical efficacy of live-attenuated RV vaccines highlights their unique capacity to concurrently induce mucosal IgA responses and systemic neutralizing antibodies, positioning them as a multiple action vector for multiple immune protection. In this review, we summarize the RV colonization of the intestine and stimulation of intestinal immunity, as well as recent advancements in RV reverse genetics, and focus on their application in the rational design of a multivalent mucosal vaccine vector targeting enteric pathogens considering the advantages and challenges of RV as a vector. We further propose molecular strategies to overcome genetic instability in recombinant RV vectors, including the codon optimization of heterologous inserts. These insights provide a theoretical foundation for developing next-generation mucosal immunization platforms with enhanced safety, stability, and cross-protective efficacy.

## 1. Introduction

The gastrointestinal tracts of humans and animals harbor extremely complex microbiota, including commensal bacteria and pathogenic bacteria. The gut microbiota are essential for host nutrient acquisition, immune development, and pathogen defense [1]. Certain commensal microorganisms within the intestinal lumen possess the capacity to transition into pathogens under specific pathophysiological conditions; such resident microbes are termed “pathobionts” [2,3]. Under homeostatic conditions, these pathobionts coexist with the host without causing detriment. However, under defined environmental triggers, these intestinal pathobionts can colonize and proliferate within small intestinal epithelial cells, precipitating clinical manifestations such as gastroenteritis and instigating inflammatory cascades that mediate more severe tissue pathology [3,4,5]. The characteristic features of enteric pathogens include the robustness of the virion and its low infectious dose. An example is rotavirus, where the feces of an infected individual can contain <10 trillion infectious particles of enteric pathogens per gram, of which only 100 particles are required to infect another person [6]. Thus, improved sanitation and hygiene have had little impact on decreasing the incidence of enteric pathogen infection. A priority of the World Health Organization (WHO) is the creation of a vaccine to combat enteric pathogen infections [7].

Enteric pathogens infections are clinically characterized by diarrhea, vomiting, fever, and severe dehydration resulting from shared pathogenic mechanisms [8,9,10]. Following colonization, these pathogens rapidly proliferate within the intestinal lumen, secreting toxins that disrupt tight junctions of the mucosal barrier within intestinal epithelial cells, dysregulate the gut microbiota, and induce epithelial apoptosis and villous atrophy [11,12,13]. In severe cases, the systemic dissemination of pathogens or toxins may trigger septic shock and multi-organ dysfunction [12]. Critically, enteric pathogens retain virulence in fecal matter, enabling fecal–oral transmission cycles and leading to persistent cross-species and environmental contamination [14]. This dual threat to One Health, which harms both animal reservoirs and human populations, demands integrated strategies for surveillance, antimicrobial stewardship, and mucosal vaccine development.

Vaccination is the preferred strategy to control infections caused by enteric pathogens. Many studies have been conducted to develop various types of enteric pathogen vaccines to control their infections, including inactivated vaccines, subunit vaccines, and live-attenuated vaccines, as well as new vaccines that have not been approved for similar vaccines, mainly including ribose nucleic acid (RNA) vaccines and virus-like particle (VLP) vaccines. Unlike inactivated vaccines, subunit vaccines, or mRNA vaccines that primarily elicit humoral immune responses through systemic antibody production, the oral live-attenuated vaccine elicits local mucosal immunity via localized replication in the intestinal epithelium [15,16,17]. This process directly stimulates mucosal-associated lymphoid tissue (MALT) to generate pathogen-specific secretory immunoglobulin A (sIgA) that neutralizes invaders at mucosal surfaces, thereby blocking colonization and transepithelial invasion [18,19,20]. Furthermore, immune cells can migrate from mucosal tissues to distant effector sites via the lymphatic system, enhancing both systemic and mucosal antigen-specific immune responses at multiple locations within the body [15,18,21,22]. The non-invasive oral administration route offers additional advantages, including cost-effective scalability, simplified cold chain logistics, and improved compliance in mass vaccination campaigns [23] (Table 1). Given the predominant fecal–oral transmission of enteric pathogens, oral vaccines targeting mucosal immunity represent a rationally designed intervention to disrupt the infection cycle at its portal of entry.

Despite the potential advantages of mucosal immunization, the hostile gastrointestinal environment characterized by acidic pH, proteolytic enzymes, and bile salts poses a significant barrier to antigen stability. Therefore, an effective oral vaccine vector must exhibit dual functionality: resistance to luminal degradation and the ability to activate intestinal mucosal immunity. RV, a fecal–orally transmitted pathogen, colonizes the small intestine, and its viral particles exhibit a high environmental stability on exposed surfaces [24]. These characteristics support the selection of rotavirus as a priority platform for global oral vaccine development. Current RV-based vectors employ virulence attenuation strategies such as targeted gene reassortment or site-directed mutagenesis to balance controlled viral replication with potent immunogenicity. However, challenges persist in maintaining genomic fidelity during large-scale production and preventing reversion to virulence, which requires innovative solutions in capsid engineering and synthetic biology.

The development of the RV reverse genetics systems represents a considerable breakthrough in virology, enabling the rational design of recombinant live-attenuated RV vectors for oral vaccine development. In 2006, the first helper virus-dependent RV reverse genetics platform was established and subsequently refined to enhance rescue efficiency (summarized in Table 2) [25,26]. A pivotal advancement occurred in 2017 when Kanai et al. engineered a plasmid-only RV rescue system that eliminated helper virus dependence and enabled the precise genomic manipulation of all 11 double-stranded RNA (dsRNA) segments [26,27]. This innovation has catalyzed RV’s adoption as a versatile vaccine chassis, with engineered strains expressing heterologous antigens from the SARS-CoV-2 spike protein, norovirus capsid, and herpes simplex virus glycoproteins demonstrating cross-protective immunity in preclinical models (Table 2). Such multivalent RV vectors leverage the virus’s intrinsic tropism for the intestinal epithelium and potent mucosal immunogenicity, offering a novel strategy to combat diverse enteric and respiratory pathogens.

In this review, we systematically summarize the characteristics and mechanisms of RV, a major intestinal pathogen, in colonizing and replicating within the intestines, the application of reassortant RV-attenuated live vaccines as an oral vaccine delivery system for mucosal vaccination, and the development of RV reverse genetics. The utility and challenges in the antigen delivery of RV reverse genetics are further evaluated. By synthesizing mechanistic insights from seminal studies, we provide a framework for optimizing RV-based multivalent vaccines that balance genetic stability, immunogenicity, mucosal immunity, and clinical safety to address the global burden of gastrointestinal infections.

## 2. Colonization of the Intestine and Stimulation of Intestinal Immunity by RVs

**Figure 1:**
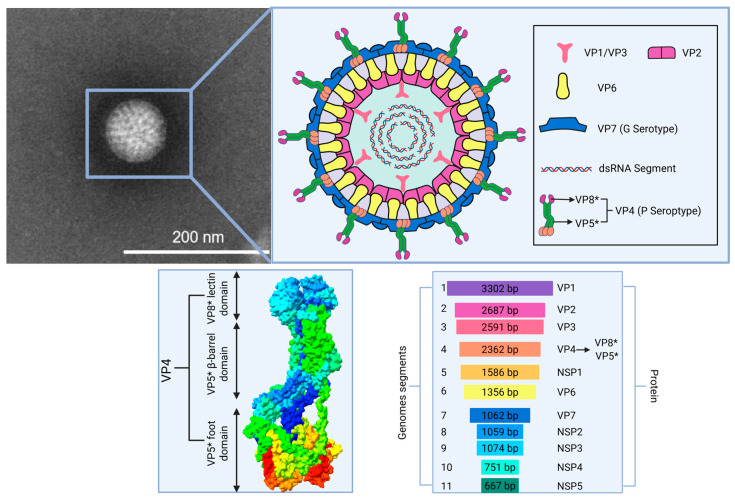
An overview of rotavirus particle structure and segmented genome. The structure of rotavirus VP4 PDB ID: 8QTZ. VP5*, subunit VP5*; VP8*, subunit VP8*.

Among viral pathogens, RV has been recognized as a major etiological agent of acute gastroenteritis, causing approximately 600,000 deaths in children under 5 years of age worldwide each year [39]. In addition, RV also causes economically significant maladies in the neonates of many domestic animals [40]. Its zoonotic transmission potential further underscores the urgency of developing cross-species protective strategies. RV members of the *Reoviridae* family are non-enveloped, icosahedral viruses with a segmented dsRNA genome encapsulated within a triple-layer capsid (Figure 1). RV demonstrates remarkable resistance to acidic and bile-rich environments, enabling survival through the harsh gastric lumen (pH 1.5–3.5) and proximal jejunum, where bile salt concentrations exceed 10 mM [41]. This resistance is orchestrated by three evolutionarily conserved mechanisms. (1) Acid-resistant capsid dynamics: structural analyses have revealed that pH-dependent conformational rearrangements in VP4 and VP7 stabilize the outer capsid and form a protease-resistant lattice that shields genomic RNA from acidic and enzymatic degradation [42,43]. (2) Accelerated intestinal transit: RV exploits clathrin-mediated endocytosis to expedite entry into the enterocyte, minimizing luminal exposure to digestive stressors [44,45]. (3) Vesicle-mediated environmental shielding: during fecal shedding, virions are encapsulated within host-derived exosomal vesicles that provide dual protection against bile salts and nucleases through lipid bilayer sequestration [46]. These synergistic adaptations spanning structural stabilization, temporal evasion, and ecological persistence enable RV to maintain infectivity throughout the fecal–oral transmission cycle.

Multiple studies now show that RV infects extraintestinal sites and tissues, including the respiratory tract (RVA RNA in nasal swabs from pigs infected with human RVA [47,48], RV replication in the salivary glands of mice and pigs [48,49] and the liver and lungs of pigs [50,51], and the pancreas, heart and other rat tissues [52]). Upon oral ingestion, the RV outer capsid protein VP4 is subjected to trypsin-mediated proteolysis in the intestinal lumen to produce VP8* and VP5* subunits. The VP8* lectin domain mediates RV attachment to different host cell receptors, including sialic acid-containing glycans, histo-blood group antigens, and integrin α2β1, depending on the virus strain [53]. The subsequent binding of VP5* with lipid raft-associated heat shock protein 70 and the clathrin-dependent endocytosis of triple-layered particles (TLPs) is triggered. Within endosomes (pH 5.5–6.0), calcium efflux induces outer capsid disassembly, and transcriptionally active double-layered particles (DLPs) are released into the cytoplasm. The viral RNA-dependent RNA polymerase (VP1) and capping enzyme (VP3) transcribe 11 capped (+) RNA segments, which are translated into structural (VP1–VP7) and nonstructural (NSP1–NSP5) proteins. Progeny genomic dsRNAs are synthesized from (+) RNA templates and assembled into nascent DLPs within viroplasms, electron-dense cytoplasmic inclusions formed by NSP2/NSP5 complexes [54]. The maturation of infectious TLPs requires NSP4-mediated DLP budding into the endoplasmic reticulum (ER), where transient envelopment facilitates the acquisition of VP4/VP7 through ER–Golgi intermediate compartment (ERGIC) trafficking. The envelope is subsequently lost during non-lytic viral egress, and the non-enveloped TLPs are released to infect adjacent enterocytes [55].

**Figure 2:**
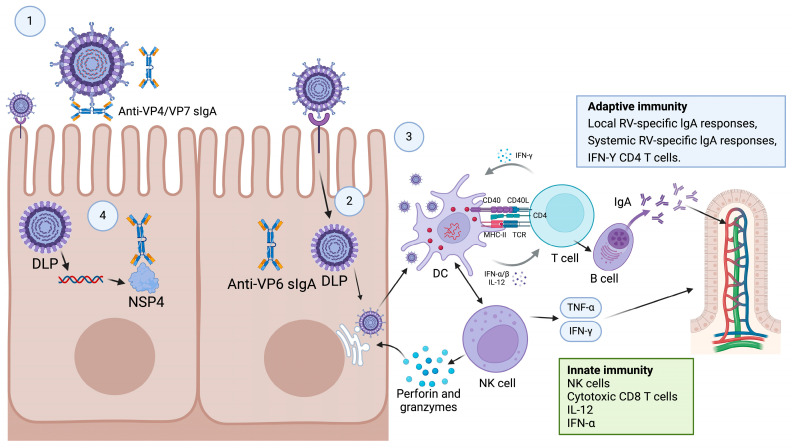
Potential mechanisms of immune response after RV infection. Intestinal RV VP4/VP7 immunoglobulin A (IgA) can prevent viral binding to enterocytes and penetration (Step 1), whereas intracellular viral replication can be inhibited by secretory anti-VP6 IgA antibody during transcytosis across enterocytes (Step 2 or 4). In addition, cytokine-secreting RV-specific T helper cells can inhibit viral replication and activate IgA antibody production by B cells (Step 3). Additionally, antibodies against NSP4 can reduce secretory diarrhea and intestinal peristalsis by inhibiting the RV stimulation of the enteric nervous system.

Naturally acquired RV infection induces immunity against RV and decreases the severity of the subsequent RV infection [56]. Within hours of rotavirus post-infection, intestinal epithelial cells (IECs) detect RV dsRNA via RIG-I/MDA5 pattern recognition receptors, which subsequently trigger the IRF3/NF-κB-mediated production of type III interferons (IFN-λ) and the upregulation of interferon-stimulated genes (ISGs) like MX1 and OAS1. This innate response recruits immune cells to the lamina propria to enhance antiviral defenses and directly inhibits viral replication [57]. Concurrently, RV antigens activate B cell differentiation in the mesenteric lymph nodes and Peyer’s patches, which subsequently triggers T cell-dependent class-switching that results in IgA antibody production. The IgA antibody contributes to the formation of an immune barrier on the surface of the intestinal mucosa to prevent viral invasion and the colonization of viruses [58]. In addition, the IgA antibody facilitates the excretion of RV from the body by binding to the virus. For instance, the intestinal IgA antibody against RV proteins VP4 and VP7 can inhibit virus binding to enterocytes, whereas the anti-VP6 IgA antibody can partially suppress viral replication during transcytosis by enterocytes [59]. T-cells also play a vital role in the adaptive immunity against RVs. T helper cells (Th) can be classified into various subtypes, including Th1, Th2, and Th17, which secrete distinct cytokines that regulate immune responses (Figure 2) [60].

In summary, RVs colonize the intestine via a series of processes, including adsorption, invasion, replication, and release. Simultaneously, RV infection stimulates the intestinal immune system to mount innate and adaptive immune responses characterized by the release of cytokines, the production of antibodies, and the activation of immune cells. Its rapid innate sensing to the adaptive immunological memory formation of the mucosal epithelium–immune system axis highlights RV’s dual role as a pathogen and vaccine vector and has guided the design of next-generation oral vaccines targeting enteric and systemic pathogens.

## 3. Live-Attenuated RV as a Gene Delivery Vector Involving Natural Gene Reassortment

**Figure 3:**
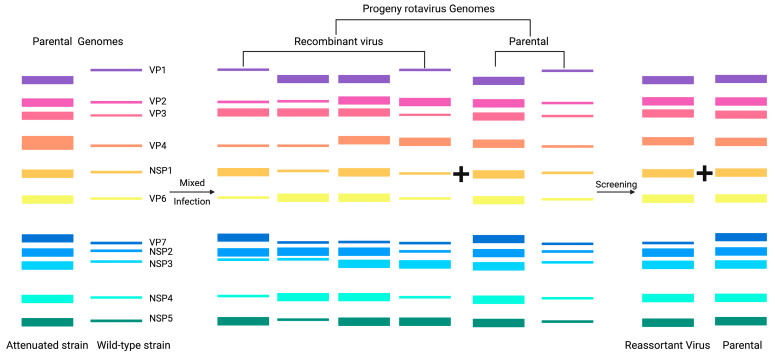
Schematic representation of reassortment in RV.

The segmented dsRNA genome of RV enables high-efficiency genetic reassortment through genetic drift between co-infecting virus strains, a versatile platform to create live-attenuated vaccine candidates [61] (Figure 3). When enterocytes are coinfected heterologous RV strains (e.g., wild-type and attenuated variants), genomic segments are randomly reassorted during progeny virion assembly to generate novel antigenic combinations. This principle has been harnessed to develop polyvalent RV vaccines with expanded serotype coverage: (1) Rotateq (pentavalent): a human–bovine reassortant vaccine that integrated VP7 (G1–G4) and VP4 (P[8]) genes from human RV strains into the bovine WC3 backbone to produce protection against predominant human RV genotypes [62]. (2) LLR3 (trivalent): the VP7 (G2, G3, and G4) genes from human RV strains were integrated into the Lanzhou lamb RV (LLR) to generate three mono-reassortant strains [63]. (3) UK-BRV: VP7 (G1–G4) or (G1–G4, G8 and G9) from human RV strains were integrated into the bovine G6P[5] UK strain to produce reassortants cross-neutralizing against heterotypic RV strains [64,65]. (4) Bivalent R191/LLR-85: a bovine RV vaccine candidate characterized by VP7 from Neonatal Calf Diarrhea Virus (G6) and backbone genes from LLR-85 (G10) that provides dual protection against a prevalent bovine RV genotype [66]. These platforms exemplify how rational reassortment can balance attenuation and immunogenicity while expanding antigenic breadth, a strategy that is extended to the insertion of heterologous pathogen antigens into RV vectors.

The development of effective polyvalent RV vaccines through combinatorial mono-reassortant strains aims to elicit broad-spectrum cross-protective immunity against currently circulating RV and future variants. However, conventional natural reassortment strategies are dependent on co-infection with two distinct RV strains at a high multiplicity of infection (MOI > 5 PFU/cell). This method suffers from a low efficiency (<15% reassortment rate) and requires a time- and labor-intensive phenotypic screening to isolate genotypes with desirable traits. These technical bottlenecks, coupled with random gene segment packaging and limited scalability, have hindered progress towards the clinical translation of RV-based vectors. Critically, natural reassortment is restricted to antigenic gene exchange within RV serotypes, and heterologous antigens cannot be inserted. Recent advances in plasmid-based reverse genetics systems have circumvented these limitations by enabling the de novo assembly of RV genomes with designer modifications. This platform permits the site-directed integration of heterologous antigen cassettes (up to 1.8 kb) into nonstructural RV genes (e.g., NSP3) while maintaining viral replication fidelity through codon optimization. Such recombinant RV vectors can simultaneously express the immunogens of multiple pathogens, which can provide a novel prophylactic or therapeutic option against intestinal pathogen infections (Table 2).

## 4. Reverse Genetic System of RV

The development of the reverse genetics (RG) system of viruses is considered to be one of the most transformative technological advances in virology. This approach leverages the viral replication cycle in which plasmids encoding wild-type and modified viral genomes are transfected into cells to produce replication-competent virions [25,27,28,29,30,31,32,33]. Reverse genetics allow for the engineering of modified viruses such as attenuated strains and viral vectors for vaccine development [25,27,28,29,30,31,32,33,67]. For RV, two principal strategies have been established: helper virus-dependent systems and helper virus-free plasmid-only systems.

### 4.1. RV Reverse Genetic System Involving Helper Viruses and Pressure Screening

**Figure 4:**
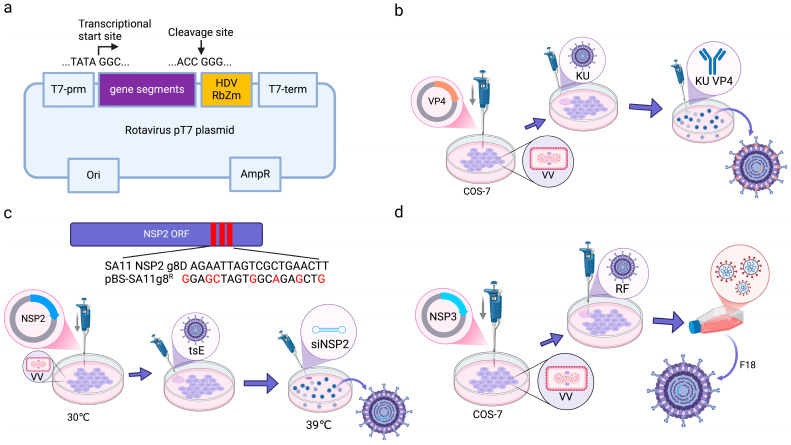
Schematic diagram of the method for the generation of recombinant RV using helper virus-dependent reverse genetics [72]. (**a**) Schematic diagram of recombinant plasmid construction. (**b**) Reverse genetic system utilizing neutralizing antibody. (**c**) Reverse genetic system utilizing temperature-sensitive mutant strain. (**d**) Reverse genetic system utilizing gene rearrangement. Image originally adapted from reference [72].

Early reverse genetics systems for RV relied on helper virus and antibody selection methods. In a seminal work in 2006, Komoto et al. engineered a T7 RNA polymerase-driven VP4 expression plasmid (Figure 4a) and transfected it into vaccinia virus (VV)-infected COS-7 cells, followed by screening using strain-specific neutralizing monoclonal antibodies [25] (Figure 4b). Recombinant RV rescue depended on high-affinity antibodies to eliminate parental helper viruses and VV-derived T7 polymerase to transcribe RV genomic segments [68]. This platform generated VP4/VP7 chimeric RV strains, which laid the groundwork for antigenically tailored vaccines [69]. However, the limitations of this method included a low rescue efficiency (<5% of transfected wells), helper virus contamination risks, and stringent antibody specificity requirements. Subsequent innovations addressed these challenges by temperature-sensitive mutants (tsE) and siRNA selection. These refinements expanded the utility of RV reverse genetics and facilitated the study of RV biology and the development of vaccines and vectors.

In 2010, Trask et al. developed a dual-selection reverse genetics platform to rescue recombinant RV-expressing modified nonstructural protein 2 (NSP2) [28]. This system introduced seven targeted mutations into the g8D locus of the RV SA11 strain to generate the recombinant plasmid pBS-SA11g8R. To isolate recombinant virions, two orthogonal selection pressures were applied: a temperature-sensitive helper virus (tsE) with restricted replication at 39 °C and siRNA targeting wild-type NSP2 mRNA (Figure 4c). In reverse genetics systems using the recombinant vaccinia virus as a helper virus, the helper virus can be removed from the rescued virus by differential temperature sensitivity or cellular tropism. This platform demonstrated unprecedented flexibility for RV genome engineering. For example, Johne utilized temperature-shift protocols to select chicken RV VP4 mutants with altered receptor tropism [30]. Navarro further expanded the use of reverse genetics by inserting heterologous sequences, including FLAG tags, hepatitis C virus E2 epitopes, and cricket paralysis virus IRES elements, into the NSP2 3′ untranslated region (UTR) to create a multifunctional RV vector for antigen delivery [70]. However, key limitations constrain broader application: the system is restricted to modifying nine RV gene segments (e.g., NSP3 and NSP4) with pre-existing temperature-sensitive mutations [71]. Due to recombination interference between selection markers, multiple genes cannot be modified at the same time. In addition, NSP2 mutations may dysregulate viroplasm formation and need to be repaired by several redundant systems to ensure the high fidelity of the DNA.

An alternative helper virus-dependent approach leverages the natural propensity of RV to selectively package rearranged genomic segments. Troupin et al. engineered a T7 RNA polymerase-driven human RV segment 7 (NSP3) expression plasmid (pBS-SA11g8R) and transfected it into COS-7 cells coinfected with the vaccinia virus (VV) and wild-type bovine RV strain RF (MOI = 30) [29]. Progeny viruses were harvested after 24 h and subjected to 18 serial passages in MA104 cells, during which the heterologous rearranged NSP3 segment outcompeted the wild-type bovine segment due to preferential packaging (Figure 4d). This strategy eliminated the need for external selection pressures (e.g., antibodies or siRNA), achieving >70% recombinant dominance by passage 15. While this system simplifies recombinant RV rescue by exploiting natural segment competition, it suffers from a low initial rescue efficiency and a protracted timeline. However, a system subjected to serial passages accumulates compensatory mutations in non-target genes, affecting viral fitness and transmissibility.

### 4.2. Entirely Plasmid-Based Reverse Genetics System for RVs

The development of plasmid-based reverse genetics systems for dsRNA viruses achieved a critical milestone in 2007, when researchers rescued orthoreoviruses using T7 RNA polymerase-expressing baby hamster kidney (BHK-T7) cells and eliminated the need for helper viruses [73]. This breakthrough catalyzed the rapid development of reverse genetics for orbiviruses and other *Reoviridae* family members and made it possible for the precise manipulation of segmented genomes to study viral replication and host interactions [74,75,76]. However, applying this approach to RVs faced unique challenges, including genomic complexity and trypsin dependency. These hurdles delayed the establishment of a fully plasmid-driven RV rescue system for over a decade until 2017, when Kanai et al. overcame these barriers by optimizing segments and incorporating heterologous trypsin during virion maturation [26].

**Figure 5:**
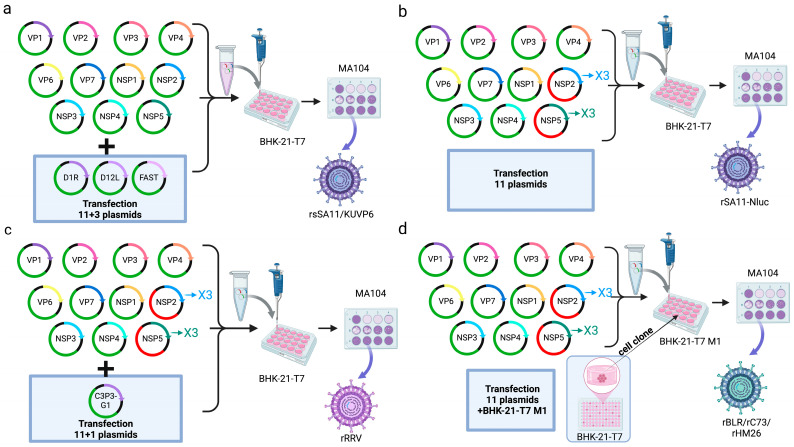
Diagram of an entirely plasmid-based reverse genetics system for RV [72]. (**a**) RV reverse genetics system based on helper plasmid fusion-associated small transmembrane protein (FAST), D1R, and D12L. (**b**) RV reverse genetics system based on three-fold the transfection amount of NSP2 and NSP5 plasmids. (**c**) RV reverse genetics system utilizing three-fold higher transfection amount of NSP2 and NSP5 plasmids and helper plasmid C3P3-G1. (**d**) RV reverse genetics system utilizing three-fold higher transfection amount of NSP2 and NSP5 plasmids and BHK-21-T7 monoclonal cell. Image originally adapted from reference [72].

In 2017, Kanai et al. achieved a seminal breakthrough in RV reverse genetics by establishing the first helper virus-free, entirely plasmid-based rescue system for the SA11 strain (Figure 5a) [26]. Based on the orthoreovirus reverse genetics platform, their RV reverse genetics system incorporated two critical innovations: a Nelson Bay orthoreovirus (NBV) fusion-associated small transmembrane (FAST) protein to enhance cell–cell fusion and genome delivery and vaccinia virus (VV)-derived capping enzymes (D1R/D12L) to ensure proper 5′ cap formation on RV transcripts. While these additions increased rescue efficiency compared with the use of the standard T7 rescue system, the overall rescue efficiency remained suboptimal, with only 1–2 wells successfully rescued per 12-well transfection [77].

To enhance rescue efficiency, Komoto et al. optimized the plasmid-based RV reverse genetics system by implementing two key modifications: dose optimization (the molar ratio of NSP2 and NSP5 plasmids was increased 3-fold relative to other RV segments during BHK-T7 cell transfection to compensate for their roles as viroplasm scaffold proteins) and minimal plasmid set (only the 11 RV genome-encoding plasmids were transfected to eliminate non-essential helper plasmids) (Figure 5b) [31]. This adjustment elevated the rescue efficiency from 8% (1/12 wells) to 50% (6/12 wells) and allowed for the recovery of low-replication-efficiency mutants such as NSP1-deficient viruses. NSP2 and NSP5 play a pivotal role in the formation of the viroplasm, where viral RNA replication and the assembly of DLPs take place [78,79,80]. This stoichiometric optimization strategy has been the basis for engineering RV vectors with antigen insertion capabilities.

Recent advancements in plasmid-based RV reverse genetics have been propelled by the engineering of the chimeric C3P3-G1 protein, which integrates two functional modules: the NP868R capping enzyme from African swine fever virus to stabilize RV transcripts via 5′-RNA guanylylation and T7 RNA polymerase from VV to amplify transcription efficiency (Figure 5c) [32]. This innovation enabled the high-titer rescue of human, murine, and simian RV strains. Based on this platform, Qin et al. further optimized the system for veterinary applications, rescuing bovine RV clinical isolates and cell culture-adapted strains within 72 h post-transfection (Figure 5d) [37]. The establishment of helper virus-free RV reverse genetics has catalyzed transformative research across pathogenic mechanisms, vector engineering, and zoonotic transmission. In particular, by enabling precise genome editing and multivalent antigen co-expression, RV reverse genetics has accelerated the design of thermostable oral vaccines with broad-spectrum coverage against evolving enteric pathogens.

## 5. Application of RV as a Vector

**Figure 6:**
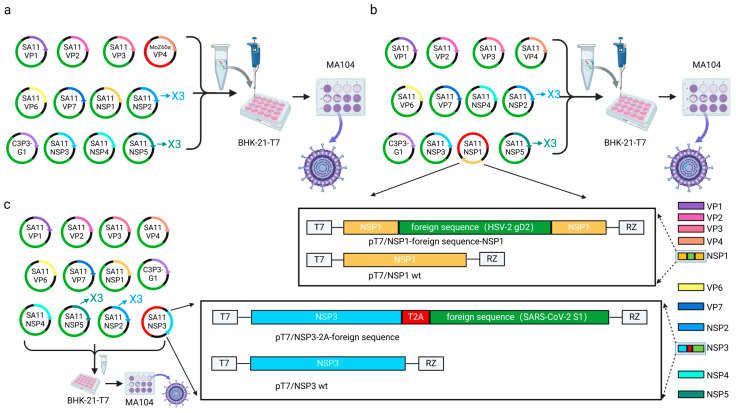
Construction strategy of RV as a transduction vector. (**a**) Construction strategy using RV gene reassortment. (**b**) Schematic presentation of the plasmids carrying the recombinant NSP1 genes for the rescue of the recombinant RV. (**c**) Modified segment seven (NSP3) plasmids used to generate recombinant RV encoding the foreign protein.

The RV reverse genetics system provides a powerful platform for the rapid development of candidate vaccines against emerging RV variants. This system enables the site-directed reassortment of specific genomic segments to generate chimeric viruses with VP4 (P-genotype) and VP7 (G-genotype) combinations derived from human and animal RV strains [33]. By replacing the VP4 or VP7 genes of animal RV backbones with those from human strains, researchers have engineered serotype-matched vaccine candidates that exhibit a high replication fidelity while maintaining attenuated virulence (Figure 6a). To date, most vaccine candidates focus on VP7 single-segment swaps due to their structural compatibility and stable packaging efficiency. In contrast, VP4-based reassortants show limited success rates and typically result in impaired replication kinetics due to mismatched interactions between heterologous VP4 spikes and native RV structural proteins. These challenges highlight that the possibility of the structure-guided rational design of proteins, such as the cryo-EM mapping of VP4-receptor interfaces and machine learning-driven codon optimization, is highly important in the generation of next-generation multivalent RV vaccines with enhanced genomic stability and manufacturability.

The RV reverse genetics system has been optimized to engineer recombinant RV strains expressing fluorescent or luminescent reporter genes for high-throughput antiviral drug screening. For example, the rsSA11-NLuc reporter virus exhibits robust luciferase activity in infected cells with a signal intensity demonstrating a dose-dependent reduction upon antiviral treatment [37,81]. Early constructs harboring reporter genes within full-length NSP1 exhibited instability during serial passaging (<5 passages). To overcome this limitation, researchers developed truncated NSP1 variants [82]. These recombinant viruses maintained genetic stability over 10 passages while expressing large reporter genes. Building on this, Hatazawa et al. inserted three distinct reporters (*Nluc*, *EGFP*, and *mCherry*) into truncated NSP1 to generate multiplex reporter RVs that simultaneously track viral replication, host cell viability, and antiviral responses in real time [83]. Parallel efforts have exploited NSP3’s modular C-terminus for reporter integration. By fusing fluorescent proteins to NSP3 via P2A self-cleaving peptides, researchers achieved the co-expression of native NSP3 and the reporter gene without compromising viral fitness, and this strategy has been validated in human, bovine, and porcine RV strains [84,85]. This approach combines real-time tractability with the capacity to deliver heterologous enteric pathogen antigens through targeted gene insertions, and it has made RV a versatile platform for the development of live-attenuated vaccine vectors.

The expression of the chimeric reporter genes in recombinant RVs has been demonstrated in intestinal tissues by the oral inoculation of the reporter viruses [86], suggesting that RV is a promising live vector for mucosal vaccination. In a seminal study, Kawamura et al. constructed a recombinant SA11 strain (rSA11-gD2) through the precise replacement of nucleotides 223-643 within the NSP1 open reading frame with the herpes simplex virus type 2 (HSV-2) glycoprotein D (gD2) gene [38]. In the 8-week-old mice inoculated with rSA11-gD2, significant increases in not only antibodies against RV but also IgG against gD2 were demonstrated, indicating that the recombinant RVs possessed excellent immunogenicity to the exogenous antigen (Figure 6b). This breakthrough highlights RV’s potential as a platform for developing vaccines against genital herpes virus and other enteric pathogens. Recent innovations extend RV’s applicability to pandemic preparedness. Philip et al. [34] and Diebold et al. [35] inserted the SARS-CoV-2 antigenic domains (including S1, NTD, RBD, ExRBD, CR, and RBM) into the NSP3 genomic segment, and they successfully achieved the expression of heterologous proteins (Figure 6c). Parallel studies by Philip et al. [87] and Kawagishi et al. [36] generated recombinant RRV (rRRV-HuNoV-VP1) expressing human norovirus (HuNoV) VP1 capsid proteins. To evaluate dual-antigen delivery, Kawagishi et al. [36] generated bicistronic gene segment 7 constructs encoding both native RV NSP3 and HuNoV structural proteins (VP1 or P domain). These replication-competent recombinants induced systemic IgG and mucosal IgA antibody responses against both RV and HuNoV in suckling mice, with demonstrable neutralizing activity to RV and HuNoV. While these findings support RV’s utility as a multivalent enteric vaccine platform, critical challenges regarding genetic stability during serial passage necessitate resolution.

To make viral vector vaccines safer, Kotaki et al. [88] developed single-round infectious RV strains through VP6 mutagenesis combined with human RV VP7 (G1/G8/G9) substitution. These replication-deficient constructs propagated exclusively in MA104-VP6 cells and caused transient diarrheal symptoms in suckling mice without generating infectious progeny. Notably, in adult mice orally immunized with the replication-deficient viruses, neutralizing antibody titers comparable to wild-type RV could be induced despite abolished viral shedding. However, VP6 modifications impair virion assembly and transcriptional fidelity and lead to reduced antigen yield and potential reversion risks during cell culture adaptation. Future development should prioritize the structure-guided optimization of VP6 mutations to preserve particle integrity while maintaining replication deficiency, as well as the exploration of alternative gene deletion strategies (e.g., non-structural protein truncations) to create second-generation single-round vectors.

## 6. Challenges of RV as a Transduction Vector

The establishment of an entirely plasmid-based reverse genetics system for RV represents an important milestone in RV research. This breakthrough technology streamlines the development of recombinant and chimeric vaccines using RV as an expression platform. However, there are still some deficiencies in the current RV reverse genetics system. For example, successfully rescued strains using this system are largely limited to a few laboratory-adapted strains that have been subcultured in vitro, and there is significant variability in the genetic stability of rescued recombinants, with frequent deletions or truncations of heterologous gene inserts. How to overcome these obstacles has become a burning question in the reliable deployment of RV as a gene delivery vector.

The genetic stability of recombinant RV strains is regulated by multiple factors, though the molecular mechanisms remain poorly elucidated. For instance, the stability of these recombinants inversely correlates with the size and sequence composition of heterologous gene inserts. Smaller heterologous sequences exhibit superior stability compared with larger counterparts. For example, the NanoLuc luciferase (NLuc; 516 bp) is more stable than the firefly luciferase (Fluc; 1653 bp) [89]. Insertions >1.1 kb exhibit 62% deletion rates after 10 passages, particularly in GC-rich sequences [87]. Notably, the codon optimization of heterologous antigens enhances genomic fidelity by 3.8-fold [90,91], implicating that nucleotide composition and secondary structure are critical determinants of stability. To advance RV as a reliable gene delivery platform, future research must prioritize the rational design of heterologous antigens and vector backbones to concurrently optimize stability, immunogenicity, and translational efficiency.

## 7. Advantages of RVs as Vectors

RV exhibits distinct advantages as a gene delivery platform, which can be summarized as follows: (1) The segmented RV genome enables facile genetic engineering to achieve the precise insertion and expression of target antigens. Given the segmented nature of the rotavirus genome, the genes encoding each segment can be rapidly reassorted in vivo by reverse genetics and accelerate the development of genotype-matched vaccine candidates. Notably, heterologous sequences exceeding 1.3 kb can be stably integrated via homologous recombination or direct cloning and broaden the applicability to multiple pathogens. (2) RV replication is confined to the small intestine to avoid the threat of genomic integration associated with other viral vectors. Furthermore, heterologous antigen insertion attenuates viral virulence and reduces adverse post-vaccination events while retaining immunogenicity. (3) Unlike vectors derived from highly pathogenic viruses, recombinant RV ensures proper folding, post-translational modification (e.g., glycosylation) and the intracellular trafficking of delivered antigens to improve vaccine safety and efficacy [91]. (4) RV is efficiently propagated in mammalian cell cultures, and high-titer yields can be obtained under biosafety level 2 (BSL-2) conditions. This scalability reduces production costs and facilitates rapid, large-scale vaccine deployment. (5) RV preferentially infects intestinal epithelial cells, and the robust mucosal IgA, systemic IgG, and T-cell responses can be elicited after the proliferation of RVs in epithelial cells. Such multiple immunity is critical for neutralizing enteric pathogens. (6) Live RV-based vaccines can induce rapid, robust, and long-lived immunity without the need for adjuvants [92]; (7) Several live-attenuated RV vaccines that have been shown to be both safe and effective to use in very young children are also licensed for use globally or primarily in their country of origin; hence, the administration of RV-based vaccines that include other heterologous antigens could be piggybacked onto current RV immunization globally used programs [36]. Collectively, its genetic maneuverability, biosafety, and ability to induce mucosal–systemic immunity make RV a vector platform for next-generation vaccines.

## 8. Conclusions and Prospects

Infectious diseases remain one of the foremost public health challenges [93]. The mucosal immune system constitutes the primary defense barrier against enteric pathogens [18]. Live viral vector vaccines capable of eliciting mucosal immunity represent a critical strategy for neutralizing intestinal infections. RV, owing to its natural tropism for the small intestine and its capacity to induce robust mucosal immune responses, has become a highly promising gene delivery platform. Breakthroughs in RV reverse genetics systems have facilitated the study of RV biology and the development of vaccines and vectors, as well as opened novel avenues for the design of vaccines against enteric pathogens [72].

RV vectors can be engineered to express protective antigens against pathogens transmitted via the fecal–oral route, such as the Hepatitis E virus and *Helicobacter pylori*. This approach enables early immune intervention by generating mucosal antibodies to prevent the adhesion of pathogens to the intestinal mucosa surface, inhibit the movement of the pathogen in the intestinal mucosa epithelium, and reduce disease transmission. The application of RV as a transduction vector presents several unresolved challenges. One main challenge lies in maintaining stable heterologous antigen expression while preserving vector functionality. Therefore, there is a need to optimize antigen insertion strategies, including structural and genomic analyses to identify optimal insertion sites combined with targeted genetic modifications (e.g., codon optimization and promoter engineering) to enhance vector stability, mucosal immune potency, and biosafety. Pre-existing anti-RV antibodies may limit host immune responses to RV-vectored vaccines, so strategies such as capsid engineering or hybrid delivery systems must be adopted to circumvent the interference of the pre-existing antibodies. Future research could also synergize the integration of RV vectors with complementary vaccine platforms (e.g., mRNA or nanoparticle-based systems) to enhance the immunogenicity of heterologous antigens and broaden protective coverage. Although RV vectors demonstrate favorable safety profiles in preclinical studies, longitudinal surveillance is imperative, particularly in high-risk populations such as infants and immunocompromised individuals [94].

Though live-attenuated RV vaccination has substantially reduced the occurrence and severity of RV-related gastroenteritis and mortality among infants and young children in high-income countries, its vaccine impact and estimated effectiveness are lower in low- and middle-income countries (LMICs) [95]. LMICs frequently encounter a constellation of complex challenges, including suboptimal sanitation elevating the pathogen burden, which potentially interferes with vaccine-induced immunity; prevalent malnutrition compromising normal immune system development and function, thereby weakening a host’s capacity to mount an effective vaccine response; and deficiencies in cold chain infrastructure, leading to reduced vaccine potency during storage and distribution [96,97,98]. Addressing these barriers necessitates a multipronged strategy encompassing vaccine refinement, systemic support, and mechanistic innovation. Essential vaccine optimizations include incorporating immunostimulatory adjuvants into oral formulations to enhance immunogenicity in immunocompromised hosts; leveraging genetic engineering to develop strains/vectors with improved bile acid tolerance, pH resistance, protease stability, or competitive gut colonization capacity; and designing vectors enabling efficient replication/antigen delivery in dysbiotic or inflamed intestinal environments. Concurrently, cold chain systems require fortification through international technical support to ensure thermostability and product integrity. Furthermore, implementing Water, Sanitation, and Hygiene interventions, anthelmintic therapies, or targeted micronutrient supplementation (e.g., zinc and vitamin A) pre-/co-vaccination may ameliorate enteric health and potentiate immunogenicity. Critically, deeper mechanistic investigation must decipher the host immune recognition of RV vectors and heterologous antigens alongside the expression kinetics and resultant immune response profiles of vectored antigens; such insights will enable the rational design of durable, broad-spectrum vaccines tailored to specific immunological phenotypes or age cohorts, ultimately advancing population-level immunological defenses against rotaviral disease.

Safety constitutes an imperative prerequisite prior to authorizing the deployment of any RV vaccine derived from reverse genetics-modified viruses in human populations [99]. Although contemporary studies indicate favorable safety profiles for recombinant rotavirus vectors, their long-term sequelae necessitate sustained surveillance and rigorous evaluation, particularly in high-risk cohorts such as infants and immunocompromised individuals. Progress necessitates a dual focus: advancing translational research through intensified preclinical and clinical investigations to comprehensively assess the feasibility and immunogenicity/efficacy profile of rotavirus-based vaccine vectors coupled with the active conduct of multicenter, large-scale clinical trials to generate robust real-world evidence and practical experience, thereby establishing a more substantial evidentiary foundation for widespread implementation and accelerating the translational trajectory from research to clinical application. Concurrently, researchers should refine the safety assessment framework for rotavirus vaccine vectors, encompassing the implementation of longitudinal safety surveillance and early warning systems for potential adverse events alongside establishing more comprehensive and granular pharmacoepidemiologic monitoring systems. Furthermore, elucidating the complex interactions between the rotavirus vector and the host immune system, as well as investigating potential adverse reactions, is imperative. A sustained emphasis on long-term safety surveillance and the identification of potential risks is paramount to ensuring the safety and reliability of rotavirus vaccine vectors during large-scale deployment, thereby providing enhanced public health safeguards for their broad application.

Although the amplification of RV seed stocks enables research within lower-risk BSL-2 facilities [100], laboratory personnel nevertheless face significant biosafety hazards in the event of improper handling. Inadequate personal protective equipment (PPE) heightens the risk of researcher exposure during procedures, potentially through the inhalation of aerosols containing recombinant RV or via accidental contact with infectious samples [101]. For the general populace, the improper disposal of laboratory waste resulting in the environmental release of recombinant RV-containing samples poses a threat; such contamination of water sources or soil could lead to the infection of healthy individuals through contact with contaminated environments or the ingestion of contaminated foodstuffs. Furthermore, genetic recombination between released recombinant RV and circulating wild-type strains in the environment holds the potential to generate novel viral variants capable of evading vaccine-induced immunity, thereby significantly complicating public health control measures. Consequently, to mitigate risks to laboratory personnel and safeguard public health, facilities handling recombinant rotaviruses must conduct rigorous, comprehensive risk assessments and implement infrastructure, engineering controls, and protective protocols strictly commensurate with mandated biosafety level standards.

In conclusion, RV has emerged as a highly promising oral vaccine vector for preventing enteric infections. The refinement of reverse genetics platforms will allow for the precise engineering of RV vectors to optimize their genetic stability and antigen immunogenicity. Elucidating molecular interactions between heterologous antigens and RV components, such as capsid proteins or replication machinery, will enable the rational design of vaccines that induce potent and pathogen-specific mucosal immunity. By addressing current limitations (e.g., insertional instability and pre-existing immunity) while leveraging RV’s advantages (e.g., intestinal tropism and scalable production), RV-based vectors are holding promise to change preventive strategies against gastrointestinal pathogens.

## Figures and Tables

**Table 1:** Comparative advantages of vaccine modalities.

Immune Strategy	Inactivated Vaccines	Subunit Formulations	Oral Live-Attenuated Vaccines
Immune response	Humoral (Th2-biased)	Humoral (Th2-biased)	Mucosal IgA antibody + Systemic Th1/Th17
Duration	Short duration	Short duration	Long-term
Cross-Protection	Limited (<30% serotype coverage)	None	Broad (>80% heterotypic immunity)
Safety concerns	Few	Few	Slight
Production	Easier	Easier	Challenging
Storage	Refrigeration	Refrigeration	Refrigeration

**Table 2:** Development of rotavirus reverse genetic system.

Year	Virus	Cell Line	Helper Virus	Helper Protein	Vector	Heterologous Proteins	Reference
2006	KU	COS-7	KU	KU VP4 antibody	/	SA11 VP4	[25]
2010	KU	COS-7	tsE	NSP2 g8D siRNA	/	SA11 NSP2	[28]
2010	RF	COS-7	RF	/	/	Human NSP3	[29]
2015	SA11	BSR5/T7	tsA	VP4 siRNA	/	Chicken VP4	[30]
2017	SA11	BHK-T7	/	FAST, D1R, D12L	/	/	[26]
2018	SA11	BHK-T7	/	NSP2, NSP5	/	/	[31]
2020	SA11	BHK-T7	/	C3P3-G1	/	/	[32]
2020	SA11	BHK-T7	/	C3P3-G1	/	Human VP4, VP7, VP6	[33]
2021	SA11	BHK-T7	/	C3P3-G1	NSP3	SARS-CoV-2 S1	[34]
2022	SA11	BHK-T7	/	NSP2, NSP5, D1R, D12L	VP4	SARS-CoV-2 S1	[35]
2022	RF	BHK-T7	/	NSP2, NSP5, D1R, D12L	NSP3	SARS-CoV-2 S1	[35]
2023	SA11	BHK-T7		C3P3-G1	NSP3	HuNoV VP1	[36]
2024	BLR	BHK-T7 M1		/	/	/	[37]
2024	C73	BHK-T7 M1	/	/	/	/	[37]
2024	HM26	BHK-T7 M1		/	/	/	[37]
2024	SA11	BHK-T7		C3P3-G1	NSP1	HSV-2 gD	[38]

## Data Availability

The raw data supporting the conclusions of this article will be made available by the authors on request.
